# Comprehensive population-wide analysis of Lynch syndrome in Iceland reveals founder mutations in *MSH6* and *PMS2*

**DOI:** 10.1038/ncomms14755

**Published:** 2017-05-03

**Authors:** Sigurdis Haraldsdottir, Thorunn Rafnar, Wendy L. Frankel, Sylvia Einarsdottir, Asgeir Sigurdsson, Heather Hampel, Petur Snaebjornsson, Gisli Masson, Daniel Weng, Reynir Arngrimsson, Birte Kehr, Ahmet Yilmaz, Stefan Haraldsson, Patrick Sulem, Tryggvi Stefansson, Peter G. Shields, Fridbjorn Sigurdsson, Tanios Bekaii-Saab, Pall H. Moller, Margret Steinarsdottir, Kristin Alexiusdottir, Megan Hitchins, Colin C. Pritchard, Albert de la Chapelle, Jon G. Jonasson, Richard M. Goldberg, Kari Stefansson

**Affiliations:** 1 Department of Internal Medicine, Stanford Cancer Center, 875 Blake Wilbur Drive, Stanford, California 94305-5826, USA; 2 Department of Internal Medicine, The Ohio State University Comprehensive Cancer Center, 460West 10th Avenue Columbus, Ohio 43210, USA; 3 University of Iceland, Sæmundargata 2, 101 Reykjavík, Iceland; 4 deCODE genetics/Amgen, Sturlugata 8, 101 Reykjavik, Iceland; 5 Department of Pathology, The Ohio State University Comprehensive Cancer Center, 460 West 10th Avenue Columbus, Ohio 43210, USA; 6 Landspitali University Hospital, Hringbraut, 101 Reykjavik, Iceland; 7 Aalborg Universitets hospital, 9000 Aalborg, Denmark; 8 Netherlands Cancer Institute—Antoni van Leeuwenhoek (NKI/AVL), Plesmanlaan 121, 1066 CX Amsterdam, The Netherlands; 9 Hvidovre Hospital, Kettegård Allé 30, 2650 Hvidovre, Denmark; 10 Mayo Clinic, Department of Internal Medicine, 5881, E Mayo Blvd, Phoenix, Arizona 85054, USA; 11 Icelandic Cancer Registry, Skogarhlíð 8, 105 Reykjavík, Iceland; 12 University of Washington, 1959 NE Pacific Street, Seattle, Washington 98195, USA; 13 West Virginia University Cancer Institute, Department of Internal Medicine, 1805 Health Sciences Center South Morgantown, 1959 NE Pacific Street, West Virginia 26506, USA

## Abstract

Lynch syndrome, caused by germline mutations in the mismatch repair genes, is associated with increased cancer risk. Here using a large whole-genome sequencing data bank, cancer registry and colorectal tumour bank we determine the prevalence of Lynch syndrome, associated cancer risks and pathogenicity of several variants in the Icelandic population. We use colorectal cancer samples from 1,182 patients diagnosed between 2000–2009. One-hundred and thirty-two (11.2%) tumours are mismatch repair deficient per immunohistochemistry. Twenty-one (1.8%) have Lynch syndrome while 106 (9.0%) have somatic hypermethylation or mutations in the mismatch repair genes. The population prevalence of Lynch syndrome is 0.442%. We discover a translocation disrupting *MLH1* and three mutations in *MSH6* and *PMS2* that increase endometrial, colorectal, brain and ovarian cancer risk. We find thirteen mismatch repair variants of uncertain significance that are not associated with cancer risk. We find that founder mutations in *MSH6* and *PMS2* prevail in Iceland unlike most other populations.

## Abstract (web summary)

Lynch syndrome is characterized by predisposition to colorectal cancer and mutations in genes involved in mismatch repair. Here, the authors use whole genome sequencing and immunohistochemistry of mismatch repair proteins to show a high prevalence of Lynch syndrome in the Icelandic population.

Lynch syndrome (LS) is the most common inherited cause of colorectal cancer (CRC). The estimated population frequency is 1:370 to 1:2,000 in Western populations^1^,^2^. LS is also associated with increased lifetime risk of several other cancer types including endometrial and ovarian cancer^3^,^4^. LS prevalence has never been determined across an entire nation.

Microsatellite instability (MSI) and mismatch repair deficiency (dMMR) are hallmarks of LS-related CRC. About 15% of CRC exhibit dMMR^5^ with 2–3% caused by germline mutations in the *MLH1*, *MSH2*, *MSH6*, *PMS2* or *EPCAM* genes^6^ while 12% of CRC cases have somatic inactivation of *MLH1* via promoter hypermethylation (*MLH1*-hm)^7^. Furthermore, double somatic mismatch repair (MMR) mutations may explain up to 67% of dMMR CRC cases without LS or *MLH1*-hm^8^.

Several groups in Europe and the United States have recommended universal screening of CRC^9^,^10^,^11^,^12^ using MSI testing or immunohistochemistry for the MMR proteins to identify potential LS cases. *MLH1*-hm can be assessed directly or inferred by the presence of a somatic *BRAF* V600E mutation^13^.

Due to the low frequency of individual LS mutations and heterogeneity in phenotypic expression, it has proven difficult to accurately establish population-based prevalences and to assess the cancer penetrance of LS gene mutations. Furthermore, numerous reported variants of uncertain clinical significance (VUS) complicate genetic counseling. Recently, a collaborative effort was undertaken to reclassify MMR variants in the International Society for Gastrointestinal Hereditary Tumours (InSiGHT) database (http://insight-group.org/variants/database/)^14^.

The Icelandic population offers several advantages for studying the genetic epidemiology and associated cancer risks of LS^15^. These include (i) a nationwide cancer registry dating back to 1955; (ii) universal tumour banking since 1935; (iii) documentation of the entire population's genealogy over centuries; and (iv) the isolation and relative homogeneity of the population which enhances the potential to discover founder mutations. To date, the DNA of over 150,000 Icelanders has undergone whole-genome analysis on microarray platforms. Additionally, 8,453 Icelanders have undergone whole-genome sequencing (WGS). Using long-range phasing, these sequence variants, down to a frequency of <0.01%, have been imputed into the genomes of those genotyped and into un-genotyped close relatives identified via genealogic databases (familial imputation). This familial imputation allows the inclusion of genotypes for cancer cases that were diagnosed decades ago, greatly enhancing the power of this study.

The objective of this study was to investigate the prevalence of LS in the Icelandic population and to establish the different etiologies for dMMR in a population-based CRC cohort. Tumours from all CRC cases diagnosed from 2000–2009 were screened for dMMR using immunohistochemistry and methylation assays and patients were genotyped for MMR variants extracted from the deCODE database. Unexplained dMMR cases underwent germline WGS and if still unexplained, tumour ColoSeq. Furthermore, variants extracted from the deCODE database were merged with the cancer registry to estimate the cancer penetrance of LS gene mutations and cancer association of VUS's found in the Icelandic population. We find that *MSH6* and *PMS2* mutations prevail in the population with a LS prevalence of 1 in 226, the highest reported so far.

## Results

### LS founder mutations in the Icelandic population

Association studies, using WGS data in the Icelandic population and information on all cancer types, revealed three LS mutations that showed significant association with CRC and endometrial cancer (Table 1). All three mutations are present at allelic frequencies >0.05%, presumably reflecting a founder effect in the population. One mutation in *MSH6* (p.Leu585Pro, class 3 per InSiGHT) and another in *PMS2* (p.Pro246Cysfs*3, class 5 per InSiGHT) were found in 9 and 12 patients with dMMR CRC, respectively (see below). A further mutation in *PMS2* (p.Met1?; pathogenic mutation in National Center for Biotechnology Information (NCBI) ClinVar^16^) was identified in the population but not in any CRC patients diagnosed during 2000–2009. These mutations were imputed and the prevalence of LS in the Icelandic population determined to be 0.442% or one in 226 individuals. The imputation quality was tested by direct genotyping of the three variants. The concordance between imputed and directly measured genotypes was 1.00 for *PMS2* p.Pro246Cysfs*3 and *PMS2* p.Met1? and 0.99 for *MSH6* p.Leu585Pro (Supplementary Table 1).

### Patient characteristics

A total of 1,208 patients were diagnosed with colorectal carcinoma in Iceland from 2000–2009, with 1,182 (97.8%) included in this study. Patient characteristics are shown in Table 2. All patients with abnormal immunohistochemistry and 78.2% of patients with normal immunohistochemistry had germline DNA available for genotyping (80.6% of the cohort).

### LS and dMMR in the CRC cohort

MMR immunohistochemistry was abnormal in 132 patients (11.2%; Table 3). *MLH1*-hm was found in 90 cases (7.6% of the cohort). Twenty-one patients with abnormal immunohistochemistry and six patients with normal immunohistochemistry had LS mutations (Table 1 and Supplementary Table 2). Eight different LS mutations were identified, including the *MSH6* p.Leu585Pro and *PMS2* p.Pro246Cysfs*3 mutations described above, four private mutations (found in a single family each) and two mutations in *PMS2*, not found in other family members or the population (*de novo* or very recent). The prevalence of LS in the CRC cohort was therefore 2.3% (27/1182). The median age at CRC diagnosis of patients with the *MSH6* p.Leu585Pro mutation was 60 (Q1 51, Q3 73; range 41–75) and the median age of patients with the *PMS2* p.Pro246Cysfs*3 mutation was 60.5 (Q1 50.5, Q3 70; range 31–86). One patient with PMS2 tumour loss had a class 3 *PMS2* variant (p.Glu705Lys). Tumour ColoSeq identified the same variant as well as likely loss of heterozygosity (LOH) in the tumour (Table 4). We, therefore, believe this variant is pathogenic and classified this case as having LS. Tumour ColoSeq was performed on 5 of 6 tumour samples from patients with LS who had normal MMR IHC (Table 4). Second hits were found in 3 cases with a *MSH6* p.Leu585Pro mutation but not 2 cases with a *PMS2* p.Pro246Cysfs*3 mutation. Twenty-one patients with dMMR tumours had neither a LS mutation or *MLH1*-hm. Sixteen patients were found to have double somatic MMR mutations by ColoSeq tumour testing (1.4% of the cohort; Table 4). Five dMMR cases remained unexplained, three cases had one somatic *MSH2* mutation and tested negative for *MSH2* methylation suggesting an unidentifiable *MSH2* mutation in germline or tumour DNA. Two cases failed tumour testing due to low tumour DNA content. None of these patients had a convincing family history of cancer. By using the 953 individuals (80.6% of the cohort) that had germline genotyping as a denominator, the sensitivity and specificity of IHC in detecting LS was 77.8% and 97.7%, respectively. The positive and negative predictive value of abnormal IHC predicting LS was 50.0% and 99.3%, respectively.

### Origin of LS mutations

**Figure 1:**
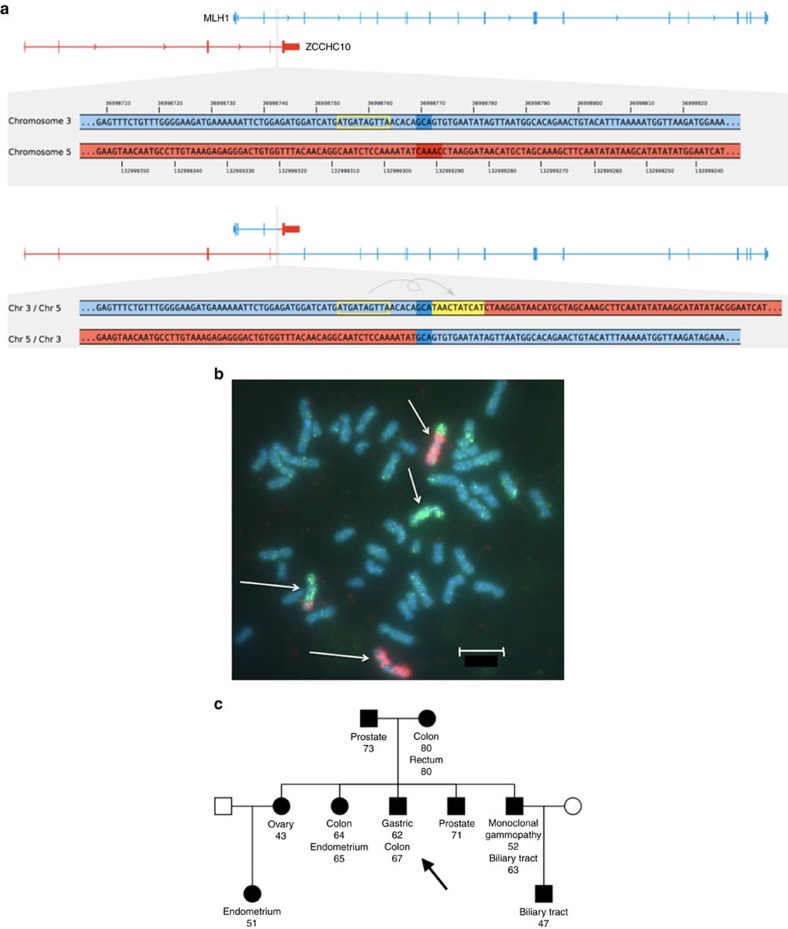
Novel *MLH1* translocation. (**a**) The top half of the figure displays enlarged GRCh38 reference sequences from within *MLH1* on chromosome 3 (blue) and from the reverse complement of chromosome 5 within *ZCCHC10* (red) in a patient with CRC with MLH1/PMS2 absent on IHC. The bottom shows the translocation breakpoints observed in the sequencing data. The translocation connects an intron of *MLH1* with an intron of *ZCCHC10* in sense of the genes' orientations. At the breakpoint, a 3-bp duplication is present on both translocation haplotypes (dark blue), a 5-bp deletion is missing from both translocation haplotypes (dark red), and a 10-bp motif from nearby got inserted in reverse complemented orientation into one of the translocation haplotypes (yellow). (**b**) FISH, chromosome 3 (red CY3), chromosome 5 (green fitc). Chromosomes 3 and 5 are marked with white arrows. The scale shown is 5 μm. (**c**) Family tree with cancers and age at diagnosis. Proband is indicated with an arrow.

The *PMS2* p.Pro246Cysfs*3 mutation carriers in Iceland, share a short haplotype with individuals from Sweden, Britain and the US (Supplementary Table 3), indicating that this mutation arose from a common ancestor, previously dated back 1,625 years^17^. Three private LS mutations in the CRC cohort and one private mutation outside of the CRC cohort were identified (Table 1 and Supplementary Fig. 1). The *MSH6* mutations (p.Val282Thrfs*10, p.Phe1088Leufs*5, p.Arg1172Lysfs*5) and the *MSH2* mutation (p.Tyr815*) were traced back to ancestors in the 1700–1800s by genotyping individuals who shared haplotypes around the mutation. Interestingly, a novel balanced translocation, T(3p22;5q31), with breakpoints in *MLH1* intron 3 and *ZCCHC10*, was identified by WGS and confirmed by karyotyping and fluorescence *in situ* hybridization (FISH) in a family with high incidence of cancer (Fig. 1). This translocation was found in a parent and was inherited by several offspring with nearly 100% cancer penetrance (colorectal, endometrial, gastric, ovarian and renal cell carcinoma; three offspring developed two cancers) but not found in the 8,453 Icelanders who have undergone WGS.

### LS and associated cancer risks

The three imputed mutations (*MSH6* p.Leu585Pro, *PMS2* p.Pro246Cysfs*3 and *PMS2* p.Met1?) vary in risk for different LS-associated cancer types as shown in Table 5. The *MSH6* p.Leu585Pro mutation is associated with a nearly 50% lifetime risk of endometrial cancer and a 36% and 25% risk of CRC in males and females as well as a ∼12% lifetime risk of brain cancer (glioma). The two *PMS2* mutations are associated with a significantly increased risk of endometrial, colorectal and ovarian cancer, although the overall cancer risks are lower than for the *MSH6* mutation. Risks for other cancers were not significantly increased and are shown in Supplementary Table 4.

### Variants of uncertain significance

We identified 13 MMR gene variants previously described as VUS's and ten novel variants, in the Icelandic population. These were imputed and odds ratios for CRC estimated. None of these variants were associated with cancer risk or unexplained dMMR suggesting they are benign variants. These variants are listed in Table 6.

## Discussion

In this comprehensive nationwide study, WGS and imputation were combined with dMMR screening of CRC to accurately determine the prevalence of LS in the Icelandic population. LS causes 2.3% of all CRC in Iceland which is similar to the prevalence in two CRC population-based studies from Finland^19^ and the US^6^ but it is higher than LS prevalence in Southern Europe^20^,^21^,^22^,^23^. The distribution of mutations among the MMR genes in Iceland is unique. LS is predominantly caused by mutations in *MSH6* and *PMS2* which are responsible for 96% of all LS cases in the CRC cohort while mutations in these two genes cause 28% of LS-associated CRC in the US^6^. Only a single mutation in *MSH2* and a single novel *MLH1* translocation were found in the population.

The low rates of *MLH1* and *MSH2* mutations constitute a negative founder effect, that is, the founders may not have introduced *MLH1* and *MSH2* mutations into the population. Genetic drift, which is more common in smaller populations, may also have influenced low rates of *MLH1* and *MSH2* mutations. Furthermore, the higher cancer penetrance and earlier onset associated with *MLH1* and *MSH2* mutations may have impacted reproductive fitness. Over 50 mutations showing founder effects have been described across the world and in some populations they have a large effect on the LS gene distribution^24^. In Finland, two *MLH1* mutations cause >50% of all LS cases^19^,^25^ and in the Netherlands^26^ and Sweden^27^, *MSH6* mutations are unusually highly prevalent. The *PMS2* p.Pro246Cysfs*3 mutation in Iceland shares a haplotype with cohorts from Sweden, US and Britain^17^. This mutation was previously dated back to around 1,625 years ago^17^, a time before Iceland was settled and it is likely to have entered the gene pool via one of the original settlers. The *MSH6* p.Leu585Pro and *PMS2* p.Met1? mutations have been described in InSiGHT and NCBI ClinVar but are not known to be founder mutations in any population. The *MSH6* p.Leu585Pro mutation is clearly pathogenic in this study with a strong cancer risk association. The *PMS2* p.Met1? mutation has a cancer risk association indicating that it is a pathogenic mutation. In addition, four private LS mutations were found and traced back to a single common ancestor in each family who lived in the 1700–1800s.

The *MLH1* germline translocation described is to our knowledge the first case of a translocation causing LS. The translocation is passed to offspring and affected family members had close to 100% cancer penetrance. Screening for translocations where LS is suspected and germline mutations cannot be found should be considered.

The population-based prevalence of LS has been estimated in CRC and endometrial cancer studies in several countries but no country has determined the prevalence empirically. It has been postulated that LS prevalence is higher than estimated in most studies^1^ and here we show the highest prevalence so far of 0.442% or 1:226 individuals. Of note, the prevalence is higher than elsewhere even though the proportion of LS in CRC is just 2.3%. This is due to the markedly low cancer penetrance of the *MSH6* and *PMS2* mutations in Iceland as compared with cancer penetrance with *MLH1* and *MSH2* mutations, dominating in most populations.

One of the strengths of this study is the ability to determine cancer risk for each of the imputable mutations. This will help tailor genetic counseling in Iceland, specific to the individual mutation. The *MSH6* p.Leu585Pro mutation is associated with a high (∼50%) lifetime risk of endometrial cancer while the *PMS2* mutations (p.Pro246Cysfs*3 and p.Met1?) have a lower lifetime risk of cancer. One of the limitations of this study is the small size of the population, which makes it difficult to estimate the risk of rare cancers such as brain cancer (confidence intervals around odds ratios are wide).

Our two-pronged approach, of linking population-based MMR variants to a dMMR CRC phenotype, imputing these variants into the population and performing an association analysis with the cancer registry, strengthens the assumption of pathogenicity (or lack thereof). The *MSH6* p.Leu585Pro mutation is described as a class 3 VUS in InSiGHT but, based on our data, should be reclassified as a pathogenic mutation. Thirteen other VUS's were not associated with a dMMR phenotype or increased cancer risks. Some of these variants can be reclassified as benign variants based on our data (variants with high population frequency and low odds ratios with narrow 95% confidence interval while others may require additional data before reclassification).

The incidence of dMMR via immunohistochemistry (11.2%) was lower than reported in many studies. In this study, an etiology was found for nearly all dMMR cases, leaving very few unexplained dMMR cases (only 0.4% of all cases remained unexplained). As 80% of all cases were genotyped, sensitivity and specificity could be calculated for the immunohistochemistry method. In three cases with the *MSH6* p.Leu585Pro germline mutation, the stains were weak but present in >1% of cells (Supplementary Table 2) and a second tumour mutation was found on ColoSeq (Table 4) indicating that these tumours developed as a result of LS. In two *PMS2* p.Pro246Cysfs*3 cases with strong stains ColoSeq did not detect a second tumour mutation so these patients developed a sporadic CRC unrelated to their germline mutation. Sporadic tumour development is likely more common with the lower penetrance mutations in *MSH6* and *PMS2*.

The population-based incidence of double somatic MMR mutations in CRC is described in this study for the first time. In total, 1.4% of all CRC had double somatic MMR mutations. These cases followed the more traditional pattern of MMR mutations with 81.3% of the mutations occurring in *MLH1* and *MSH2*. The cancer etiology in three cases with just one somatic hit in *MSH2* remains unclear. The family history was unconvincing so it is unlikely that a LS mutation was missed. Nevertheless such cases present a challenge for genetic counseling. The tumour location in LS patients was left-sided or rectal in ∼50% of cases while it was predominantly right-sided in the *MLH1*-hm and double somatic MMR cases (>80%). It is possible that left-sidedness is more common with *MSH6* and *PMS2* mutations as compared with *MLH1* and *MSH2* mutations. Less than 10% of patients with dMMR CRC presented with metastastic disease, similar to other studies on dMMR CRC. A male predominance was seen in the LS group, a female predominance in the *MLH1*-hm group while the double hit sporadic tumours had a more even gender distribution.

In conclusion, this study has mapped the prevalence of LS in the Icelandic population, and its associated cancer risks and established that one in 226 Icelanders carry this syndrome. Furthermore, the contribution of different etiologies to dMMR CRC has been described. It is likely that among the population of 320,000 Icelanders, >1,000 individuals carry mutations causing LS. Identifying these individuals and establishing a cancer screening program are imperative next steps. Thirteen class 3 variants were not associated with an increased cancer risk and one class 3 variant was determined to be pathogenic; these results could guide counseling of patients with these variants. Finally, we have shown that a combined approach of phenotypic screening and population-based WGS can be used to extensively map out an inherited syndrome with a well-recognized phenotype.

## Methods

### CRC patient population

**Figure 2:**
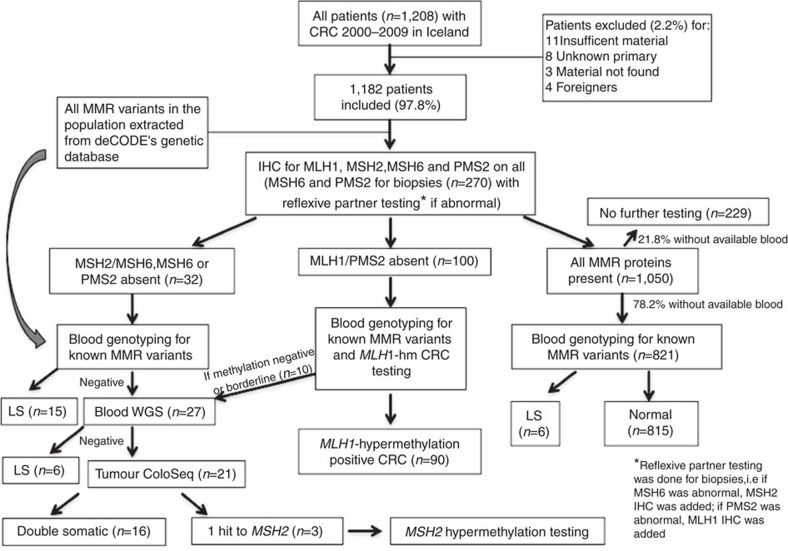
Study design. CRC, colorectal cancer; IHC, immunohistochemistry; LS, Lynch syndrome; WGS, whole-genome sequencing.

All patients diagnosed with CRC in Iceland from 1 January 2000 to 31 December 2009 as identified through the Icelandic Cancer Registry (http://www.krabbameinsskra.is) were included in the study. Figure 2 depicts the study design and reasons for exclusion. The Icelandic National Bioethics Committee (VSNb2013010033/03.15), the Icelandic Data Protection Authority (2013010109TS), and the Ohio State University (OSU) Institutional Review Board (2013C0144) approved this study. Descriptive statistics (median with quartiles for age and frequency for categorical variables) were provided to summarize the patient population. Cases where the origin of the primary tumour was regarded to be non-colonic (appendix, ileum and so on.) or unknown were excluded. Charts were accessed and clinical information obtained. Foreigners and patients without available tumour material were excluded. In cases of synchronous CRC, only the tumour with the most advanced stage or stage subdivision was analysed. In cases of metachronous CRC, the tumour diagnosed in the defined period was used (the first one if there were two).

### Immunohistochemistry and *MLH1* hypermethylation testing

Haematoxylin and eosin stained tumour slides (obtained from Landspitali University Hospital, Akureyri Hospital and Histopathology Institute, Sudurlandsbraut) were reviewed and formalin-fixed paraffin embedded tissue blocks selected for further analysis. Tissue microarrays were built at the OSU Pathology Core Facility with two 1.0 mm cores from each case. Immunoperoxidase staining was performed using primary antibodies for MLH1 (Novacastra, Buffalo Grove, IL; NCL-L-MLH-1; Clone:ESO5; diluted 1:500), MSH2 (Calbiochem, [Merck Biosciences AG], Basel-Land, Switzerland; NA-27; Clone:FE11; diluted 1:3,000), MSH6 (Epitomics Inc, Burlingame, CA; AC-0047; Clone:EP49; diluted 1:800) and PMS2 (BD Pharmingen, San Jose, CA; 556415; clone:A16-4; diluted 1:300). Stains were scored as present with convincing nuclear staining in tumour cells with a positive internal control. In cases where only biopsies were available or rectal cancer cases where pre-radiation therapy biopsy samples were chosen, biopsies were stained for MSH6 and PMS2. If either were lost, stains were performed for MLH1 (if PMS2 lost) and MSH2 (if MSH6 lost). In cases scored as absent where no *MLH1*-hm or germline mutation was found, stains were repeated on a whole section (if previously done on tissue microarrays). In cases found to have LS with normal immunohistochemistry, stains were repeated on a whole section.

Tumours with MLH1 loss were tested for *MLH1*-hm by pyrosequencing using the Pyromark Q96 CpG MLH1 kit (QIAGEN, Hilden, Germany). Up to five 1.0 mm cores were used for tumour DNA and germline DNA extraction by standard methods. Methylation levels of ≥15% were classified as positive for hypermethylation. The Pyromark Q96 CpG MLH1 kit (QIAGEN, Hilden, Germany) tests hypermethylation in four sites of the promoter region of *MLH1*, positions −209 to −188. Polymerase chain reaction is used to amplify the region and the degree of methylation of four CpG sites is analysed in a single pyrosequencing reaction by taking the average of four sites. In cases where only biopsy tissue was available and *MLH1*-hm testing failed, BRAF immunohistochemistry for the *V600E* mutation was performed as previously described using the VE1 antibody (1:700, incubation 15 min; Spring Bioscience, Pleasanton, CA) with an automated staining system (Bond Autostainer; Leica Microsystems, Buffalo Grove, IL)^28^. BRAF V600E immunohistochemistry was graded as positive for the mutation when diffuse cytoplasmic staining in tumour cells was seen.

### Detection of LS mutations

The whole genomes of 8,453 Icelanders, irrespective of cancer status, were sequenced using Illumina technology to a mean depth of at least 10 × , unveiling 31.6 million single-nucleotide polymorphisms (SNP) and short insertions/deletions that meet stringent quality criteria. These variants were imputed into 150,656 Icelanders whose DNA had been genotyped with various Illumina SNP chips and phased using long-range phasing^29^,^30^.

All patients with dMMR tumours had germline DNA genotyped for MMR variants found by WGS of the 8,435 Icelanders. If no LS mutations were identified, WGS was undertaken on blood samples with Illumina technology or, in cases where blood DNA was not available, DNA from archived formalin-fixed paraffin embedded normal tissue was subjected to Sanger sequencing of the MMR genes. Single variant genotyping was carried out by Sanger sequencing. The *PMS2* p.Pro246Cysfs*3 variant was also genotyped using PCR and size fractionation. The region around chr7:5997387 was amplified from genomic DNA from blood using conventional PCR and size fractionated on 3730 DNA Analyser (Applied Biosystems Inc.). The *PMS2* p.Met1? variant was also genotyped using the Centaurus (Nanogen) platform. All primer sequences are listed in Supplementary Table 5. To assess the quality of the imputation, a set of imputed carriers and non-carriers of the variants were genotyped using single marker assays, comparing imputed genotypes to genotypes obtained by direct genotyping (see Supplementary Table 1).

### Whole-genome genetic analyses of the Icelandic population

WGS and genotyping, imputation and association analysis used in the Icelandic population were as described^15^. The whole genomes of 8,453 Icelanders were sequenced using Illumina technology to a mean depth of at least 10 × (median 32 × ). SNPs and indels were identified and their genotypes called for all samples simultaneously using the Genome Analysis Toolkit (GATK version 2.2–13)^31^. Genotype calls were improved by using information about haplotype sharing, taking advantage of the fact that all the sequenced individuals had also been chip-typed and long range phased. A total of 31.6 million SNPs and short indels that met stringent quality criteria were identified in the 8,453 sequenced Icelanders. These variants were then imputed into 150,656 Icelanders who had been genotyped with various Illumina SNP chips and their genotypes phased using long-range phasing^29^,^30^. Genealogical deduction of carrier status of 294,212 un-typed relatives of chip-typed individuals further increased the sample size for association analysis and increased the power to detect associations. Individuals who have any form of cancer and controls were derived from the chip-typed individuals and un-typed relatives. Association testing for case–control analysis was performed using logistic regression.

To account for inflation in test statistics due to cryptic relatedness and stratification, we applied the method of LD score regression^32^,^33^. With a set of 1.1 M variants, we regressed the *χ*^2^ statistics from our genome-wide association study scan against LD score and used the intercept as a correction factor. The LD scores were downloaded from an LD score database (see URL). For the traits reported here, the estimated correction factors were 1.15 for CRC, 1.12 for colon cancer, 1.04 for rectal cancer, 1.05 for endometrial cancer, 1.04 for ovarian cancer, 1.03 for brain cancer, 1.25 for breast cancer, 1.07 for bladder cancer, 1.02 for esophageal cancer, 1.01 for gallbladder/bile duct cancer, 1.13 for gastric cancer, 1.02 for liver cancer, 1.00 for myelodysplastic syndrome, 1.09 for pancreatic cancer, 1.22 for prostate cancer, 1.01 for renal/ureteral cancer, 1.03 for testicular cancer.

### Information on cancer in the study population

Individuals affected with all forms of cancer were identified through the Icelandic Cancer Registry. The Icelandic Cancer Registry contains all cancer diagnoses in Iceland from 1 January 1955. Over 90% of diagnoses are histologically confirmed. The Icelandic Cancer Registry contains records of 4,434 Icelandic CRC patients (52% males) diagnosed from 1 January 1955 until 31 December 2013. Recruitment of cancer cases of all types was initiated in 2001 and included all prevalent cases as well as newly diagnosed cases from that time. Of the 1,685 CRC cases diagnosed from 1 January 2001–31 December 2013, 1,354 (80%) participated in our study. Patients are recruited by trained nurses on behalf of the patients' treating physicians, through special recruitment clinics. Participants in the study sign an informed consent form, donate a blood sample and answer a lifestyle questionnaire.

The median age at diagnosis for all consenting cases was 72 years, the same as that for all CRC patients in the Icelandic Cancer Registry. In addition to the chip-genotyped cases, we used information on 2,480 CRC cases without chip information whose genotype probabilities were imputed using methods of familial imputation^15^. The 262,425 controls (combined chip-typed and familially imputed) used in this study consisted of individuals from other ongoing genome-wide association studies at deCODE. No individual disease group is represented by more than 10% of the total control group. Samples from other cancer cases used in the cross-risk analysis come from other ongoing projects at deCODE Genetics. All subjects were of European ancestry.

All sample identifiers were encrypted in accordance with the regulations of the Icelandic Data Protection Authority. Approval for the study was granted by the Icelandic National Bioethics Committee (ref. VSNb201410008/03.12) and the Icelandic Data Protection Authority (ref. 2014101449).

### The Icelandic genealogy

The Icelandic genealogical database contains 819,410 individuals back to 740 AD. Of the 471,284 Icelanders recorded to have been born in the 20th century, 91.1% had a recorded father and 93.7% had a recorded mother in the database. Similarly, of the 183,896 Icelanders recorded to have been born in the 19th century, 97.5% had a recorded father and 97.8% had a recorded mother.

### Haplotype analysis for the *PMS2* frameshift mutation

Seven SNPs spanning exons 5–9 in the *PMS2* gene were analysed in patients carrying the *PMS2* p.Pro246Cysfs*3 founder mutation and compared to the haplotype found in carriers of this founder mutation in US, Swedish and British populations^17^. These are described in Supplementary Table 3.

### *MLH1* translocation, karyotyping and FISH

To scan for structural variation, all reads from the WGS data that align to the respective genes including 100 Kbp flanking regions to both sides of the genes were extracted. All reads that have a mapping quality of 0 or an average PHRED-scaled base calling quality of 25 or below were excluded. The remaining sets of reads were analysed for read pair discordance: A read was considered be part of a discordant read pair if (1) its mate is unaligned or aligns to a different chromosome or (2) the read and its mate align in unexpected orientation to each other or (3) the distance between the alignment positions of the read and its mate differ by more than three standard deviations from the mean insert size of the sequencing library. The discordant pairs that passed these filters were analysed manually for clusters where the reads align to similar positions. Suspicious clusters were further examined for alternative alignment locations of the two read ends and discarded if the location in the reference genome was repetitive. Further, clusters were discarded as common variants if present in many whole-genome sequenced controls. The only cluster that passed all filters indicates the translocation between chromosome 3 (*MLH1*) and chromosome 5 (*ZCCHC10*). The exact breakpoint positions and target-site events were inferred from 36 soft-clipped reads from the two locations on chromosome 3 and 5 in the WGS data of one patient and confirmed by 31 soft-clipped reads in a relative.

Metaphase chromosomes were harvested from PHA-stimulated (phytohaemagglutinin) lymphocytes cultured in McCoýs 5A medium with 20% fetal calf serum applying standard methods. Chromosomes were G-banded and karyotyped^34^. Whole chromosome FISH was performed with directly labelled StarFISH paint probes (Gambio Ltd., Cambridge, UK) for chromosomes 3 (CY3-labelled) and 5 (fitc-labelled) from Cambio, following their protocols. Chromosomes were counterstained with DAPI (4,6-diamidino-2-phenylindole). Image analysis was done using Leica CW4000 FISH software and Leica DMRA2 microscope with appropriate filters for FITC and Cy3.

### Somatic tumour mutation and *MSH2* methylation analysis

Three micrograms of tumour DNA (1 ug in two biopsy cases) were used to perform ColoSeq tumour next-generation sequencing in dMMR cases that remained unexplained after germline mutation and *MLH1*-hm analysis and selected LS cases^35^. ColoSeq tumour is a clinical diagnostic assay that detects single nucleotide, indel and deletion/duplication mutations in *MLH1*, *MSH2*, *MSH6*, *PMS2*, and *EPCAM* as well as the *BRAF* p.V600E mutation and phenotypic MSI. The assay uses paired-end sequencing on the Illumina HiSeq 2500 instrument to sequence all exons, introns, and flanking sequences at >300 × average coverage. LOH was determined by haplotype analysis of the variant allele fraction. Cases were considered solved if: (1) Two pathogenic or likely pathogenic mutations were identified (mutations identified as class 4 or 5 or predicted to result in protein truncation); or (2) One pathogenic or likely pathogenic mutation was identified with associated LOH. Cases were considered possibly solved if only one pathogenic or likely pathogenic mutation was identified with possible LOH. Phenotypic MSI was assessed with ColoSeq tumour next-generation sequencing data using mSINGS (MSI by NGS)^36^.

One microgram tumour DNA was treated with sodium bisulphite using the EZ Methylation Gold kit (Zymo Research, Irvine, California), according to the manufacturer's instructions. Approximately 100 ng bisulphite-converted template was tested for the presence of methylation by two methods, quantitative CpG pyrosequencing and real-time methylation-specific PCR (qMSP) and temperature denaturation analysis, alongside the Universal Methylated Human DNA Standard (fully methylated) and Human WGA Non-Methylated DNA (unmethylated) control samples (Zymo Research, Irvine, California). CpG pyrosequencing was conducted as previously described^37^ with some modifications. PCR amplification was performed with 1 μM of each primer 5′-Biotin-TTTGGAAGTTGATTGGGTGTGGT-3′ and 5′-CYACTTCTCCYACATACCCTAAAAAAAAC-3′ and 3 μM MgCl2 with cycling conditions of 95 °C for 5 min, followed by 45 cycles of 94 °C for 30 s, annealing at 66 °C for 30 s and extensions at 72 °C for 30 s, then a final extension step at 72 °C for 10 min. PCR products were prepared for pyrosequencing according to the standard protocol. Pyrosequencing was performed using internal primer 5′-CCACACCCACTAAACTATT-3′ with nucleotide dispensation order 5′-CTA CGA CTC CTC ATC GAT CCA GAT CAG ATC GAT ACA GAC ATC AGA TCA GAC AC-3′ on the PyroMark Q96-ID system (Qiagen). Methylation levels were measured using the PyroMark CpG Methylation software. The methylation levels were quantified at six consecutive CpG sites. Semi-quantitative methylation-specific PCR of the *MSH2* promoter was performed using primers 5′-GTAGTAGTTAAAGTTATTAGCGTGCGCG-3′ and 5′-TCCTTCGACTACACCGCCATATCG-3′ with cycling conditions of 95 °C for 6 min, followed by 42 cycles of 94 °C for 30 s, annealing at 59 °C for 30 s, and extension at 72 °C for 30 s, followed by a melt curve from 65 °C to 94 °C at 0.5 °C increments for 5 s each. A MyoD control reaction for DNA input and normalization of *MSH2* methylation levels was included, as we have previously described^38^. Real-time qMSP was performed on the CFX-96 Real-time System (BioRad).

### Data availability

The variants found in the MMR genes (pathogenic, likely pathogenic and variants of unknown significance) that support the findings of this study have been deposited in InSiGHT (https://www.insight-group.org/variants/databases/) with the accession codes (http://insight-database.org/variants/0000004813 to http://insight-database.org/variants/0000004866). LD score database (accessed 23 June 2015) ftp://atguftp.mgh.harvard.edu/ brendan/1k_eur_r2_hm3snps_se_weights.RDS. All other remaining data are available within the Article and Supplementary Files, or available from the authors upon request.

## Additional information

**How to cite this article:** Haraldsdottir, S. *et al*. Comprehensive population-wide analysis of Lynch syndrome in Iceland reveals founder mutations in *MSH6* and *PMS2*. *Nat. Commun.* **8,** 14755 doi: 10.1038/ncomms14755 (2017).

**Publisher's note**: Springer Nature remains neutral with regard to jurisdictional claims in published maps and institutional affiliations.

## Supplementary Material

Supplementary Information Supplementary Figures and Supplementary Tables

Peer Review File

## Figures and Tables

**Table 1:** Lynch syndrome mutations in the Icelandic population.

**Gene**	**Hg38 location**	**Coding change**	**Protein change**	Pathogenicity*	**Nr. pts in CRC cohort**	**Carrier frequency**
*PMS2*	chr7:5997387	c.736_741del6ins11	p.Pro246Cysfs*3	Class 5	12	0.234%†
*PMS2*	chr7:6009018	c.2T>A	p.Met1?	Pathogenic‡	0	0.092%†
*MSH6*	chr2:47799737	c.1754T>C	p.Leu585Pro	Class 3	9	0.080%†
*MLH1*	chr3:36998760	Balanced translocation (Fig. 1)		NK	1	1 Family§
*MSH6*	chr2:47798826	c.843_844insAC	p.Val282Thrfs*10	NK	1	1 Family§
*MSH6*	chr2:47803500	c.3261dupC	p.Phe1088Leufs*5	Class 5	1	1 Family§
*MSH6*	chr2:47804984	c.3514dupA	p.Arg1172Lysfs*5	Class 5	1	1 Family§
*MSH2*	chr2:47478506	c.2445T>A	p.Tyr815*	NK	0	1 Family†
*PMS2*	chr7:6004007	c.211_214del	p.Asn71Aspfs*4	NK	1	1 Patient§
*PMS2*	chr7:5982885	c.2113G>A	p.Glu705Lys	Class 3	1	1 Patient§

CRC, colorectal cancer; MMR, mismatch repair; NK, not known; Nr., number; pts=patients.

^*^Pathogenicity as listed in InSiGHT, if known.

^†^Extracted from deCODE's database.

^‡^Listed as pathogenic in NCBI ClinVar^16^, not listed in InSiGHT.

^§^Found by sequencing patients with dMMR CRC.

**Table 2:** Patient and tumour characteristics in the colorectal cancer cohort.

	**Proficient MMR (*****n*****=1,044)**	**Lynch syndrome (*****n***=27)*	***MLH1*****-hm (*****n*****=90)**	**Double somatic MMR (*****n*****=16)**	**dMMR unexplained (*****n*****=5)**
Age, median (Q1, Q3)	71 (61, 78)	62 (55, 72)	78 (73, 84)	69 (57, 79)	75 (35, 86)
*Gender*					
Male	601 (57.6%)	22 (81.5%)	25 (27.8%)	9 (56.3%)	2 (40.0%)
Female	443 (42.4%)	5 (18.5%)	65 (72.2%)	7 (43.7%)	3 (60.0%)
*Stage at diagnosis*					
I	193 (18.5%)	5 (18.5%)	9 (10.0%)	1 (6.3%)	3 (60.0%)
II	282 (27.0%)	16 (59.3%)	47 (52.2%)	9 (56.3%)	2 (40.0%)
III	298 (28.5%)	4 (14.8%)	24 (26.7%)	4 (25.0%)	0
IV	230 (22.0%)	2 (7.4%)	6 (6.7%)	1 (6.3%)	0
Unknown	41 (3.9%)	0	4 (4.4%)	1 (6.3%)	0
*Location*					
Right	357 (34.7%)	14 (51.9%)	76 (86.4%)	13 (81.3%)	2 (40.0%)
Left	366 (35.5%)	9 (33.3%)	8 (9.1%)	2 (12.5%)	1 (20.0%)
Rectum†	307 (29.8%)	4 (14.8%)	4 (4.5%)	1 (6.2%)	2 (40.0%)
Metachronous	30 (2.9%)	1 (3.7%)	6 (6.7%)	0	0
Synchronous	30 (2.9%)	2 (7.4%)	4 (4.4%)	0	0

dMMR, deficient mismatch repair activity; IHC, immunohistochemistry; *MLH1*-hm, MLH1 hypermethylation; MMR, mismatch repair; *N*, number; Q, quartiles.

^*^Six LS patients with CRC with normal IHC are included here.

^†^Rectosigmoid location is included with rectum.

**Table 3:** Colorectal cancer cases with abnormal mismatch repair protein immunohistochemistry.

**Absent stains on IHC**	**Etiology of mismatch repair deficiency**				
	***MLH1*****-hm**	**LS**	**Double somatic MMR**	**Unexplained**	**Nr. cases**
MLH1/PMS2	90*	1	8	1	100 (8.5%)
MSH2/MSH6	NA	2†	5	4	11 (0.9%)
MSH6	NA	7	1	0	8 (0.7%)
PMS2	NA	11‡	2	0	13 (1.1%)
Total	90 (7.6%)§	21 (1.8%)	16 (1.4%)	5 (0.4%)	132 (11.2%)

IHC, immunohistochemistry; LS, Lynch syndrome; MLH1-hm, *MLH1* hypermethylation; NA, not applicable; Nr., number.

The denominator in the table is all CRC patients (*n*=1182). The six patients with LS and normal IHC are not depicted in this table.

^*^One case had MLH1/PMS2/MSH2 missing.

^†^One case had MSH2/MSH6/PMS2 missing.

^‡^One case had MSH6/PMS2 missing.

^§^Three biopsy cases failed *MLH1*-hm testing but were positive for BRAF V600E via IHC.

**Table 4:** Somatic mismatch repair gene mutations as tested by ColoSeq.

**Study ID**	**mSINGS**	**MSI**	***MLH1*****-hm**	**Absent stains on IHC**	**Gene**	**MMR mutation no.1**	**MMR mutation no. 2**	**Outcome**
197	0.4062	MSI-H	10%	MLH1/PMS2	*MLH1*	c.493del (p.A165Lfs*2)	LOH	Double somatic
237	0.4531	MSI-H	4%	MLH1/PMS2	*MLH1*	c.2237del (p.L746Rfs*37)	LOH	Double somatic
466	0.5538	MSI-H	5%	MLH1/PMS2	*MLH1*	c.1684C>T (p.Q562*)	LOH	Double somatic
506	0.5079	MSI-H	4%	MLH1/PMS2	*MLH1*	c.199G>A (p.G67R)	LOH	Double somatic
745	0.4923	MSI-H	10%	MLH1/PMS2	*MLH1*	c.1783_1784del (p.S595Wfs*14)	LOH	Double somatic
985	0.4462	MSI-H	10%	MLH1/PMS2	*MLH1*	c.588del (p.K196Nfs*6)	c.171del; c.299G>A (p.K57Nfs*2; p.R100Q)	Double somatic
1034	0.5538	MSI-H	6%	MLH1/PMS2	*MLH1*	c.2142G>A (p.W714*)	c.670_673dup (p.R226*)	Double somatic
1141	0.4923	MSI-H	NT	MLH1/PMS2	*MLH1*	c.2206G>T (p.E736*)	LOH	Double somatic
246	0.4769	MSI-H	NT	MSH2/MSH6	*MSH2*	c.545_552del (p.D182Vfs*4)	c.1661+1G>A (Splicing)	Double somatic
1082	0.3492	MSI-H	NT	MSH2/MSH6	*MSH2*	c.82del (p.E28Rfs*36)	c.1310del (p.V437Gfs*17)	Double somatic
1049	0.4754	MSI-H	NT	MSH2/MSH6	*MSH2*	c.942+3A>T (splicing)	LOH	Double somatic
959	0.5846	MSI-H	NT	MSH2/MSH6	*MSH2*	c.2459-2A>C (splicing)	c.2352del (p.H785Mfs*27)	Double somatic
951	0.5156	MSI-H	NT	MSH2/MSH6	*MSH2*	c.425C>G (p.S142*)	c.942+3A>T (splicing)	Double somatic
509	0.1587	MSS	NT	MSH6	*MSH2*	Inversion; c.2210+7G>T (germline)*	LOH (maybe)	Possibly double somatic
1231	0.4462	MSI-H	NT	PMS2	*PMS2*	c.325delG (p.E109Kfs*3)	c.444delC; c.903+1G>A (p.Y149Tfs*52; splicing)	Double somatic
1022	0.2857	MSI-H	NT	PMS2	*PMS2*	Exon 11 homozygous deletion	LOH	Double somatic
427	0.4462	MSI-H	NT	PMS2	*PMS2*	c.2113G>A (p.E705K, germline)	LOH (likely)	Lynch syndrome†
1057	0.5692	MSI-H	NT	PMS2	*PMS2*	c.211_214del (p.N71Dfs*4, germline)	c.2113G>A (p.E705K), LOH (maybe)	Lynch syndrome†
339	0.5626	MSI-H	5%	MLH1/PMS2	*MLH1*	Germline translocation‡	c.2041G>A (p.Ala681Thr)	Lynch syndrome†
786	0.2787	MSI-H	NT	Normal (MSH6 weak, 1–5%)	*MSH6*	c.1754T>C (p.L585P, germline)	c.3509delT (p.I1170Mfs*14)	Lynch syndrome†
30	0.1228	MSS	NT	Normal (MSH6 weak, 5%)	*MSH6*	c.1754T>C (p.L585P, germline)	c.3261del (p.F1088Serfs*2)	Lynch syndrome†
94	0.4032	MSI-H	NT	Normal (MSH6 weak, 20–30%)	*MSH6*	c.1754T>C (p.L585P, germline)	c.3016T>G (p.Y1006D)	Lynch syndrome†
1250	0.1538	MSS	NT	Normal	*PMS2*	c.736_741del6ins11 (p.Pro246Cysfs*3, germline)	None detected	Lynch syndrome (sporadic tumour)†
873	0.0615	MSS	NT	Normal	*PMS2*	c.736_741del6ins11 (p.Pro246Cysfs*3, germline)	None detected	Lynch syndrome (sporadic tumour)†
162	0.5385	MSI-H	NT	MSH2/MSH6	*MSH2*	c.2458+2delT (splicing)	None detected§	Not solved
1127	0.2188	MSI-H	NT	MSH2/MSH6	*MSH2*	c.943-1G>C (splicing)	None detected§	Not solved
1108	0.4154	MSI-H	NT	MSH2/MSH6	*MSH2*	c.2131C>T (p.R711*)	c.2635-10T>G (germline, splicing)‖§	Not solved
418	0.1077	MSS	NT	MSH2/MSH6	*MSH2*	c.1077-2736T>G (deep intronic)	LOH	Not solved¶
827	0.0923	MSS	2%	MLH1/PMS2	None	Failed testing		Not solved¶
240	0.2742	MSI-H	14%	MLH1/PMS2	None	No hits to MMR genes—*BRAF* V600E		*MLH1*-hm

LOH, loss of heterozygosity; MLH1-hm, *MLH1* hypermethylation; MMR, mismatch repair; MSI-H, microsatellite unstable high; mSINGS, microsatellite instability by next-generation sequencing; MSS, microsatellite stable, NT ,not tested.

^*^c.2210+7G>T (rs374675118)—minor allelic frequency is 0.969% in the Icelandic population without CRC association (see Table 6: VUS).

^†^Two cases with *de novo* germline *PMS2* mutations (427, 1057) and the *MLH1* germline translocation (339) and five cases with normal MMR IHC underwent tumour Coloseq testing to confirm a second hit.

^‡^See Fig. 1.

^§^Negative for *MSH2* hypermethylation.

^‖^Not clearly pathogenic.

^¶^Indeterminate study due to low tumour content.

**Table 5:** Icelandic Lynch syndrome mutations and odds ratios for different cancers.

	**Cancer type**	**Odds ratio**	**95% Confidence interval**	***P*****-value**	**Cases**	**Controls**	**Info**	**Cancer risk (M)**	**Cancer risk (F)**
*MSH6* p.Leu585Pro Pop carrier freq: 0.080%	Endometrial cancer	32.8	13.5–80.0	1.61 × 10^−14^	923	115,104	0.92	NA	49.2%
	Colorectal cancer	10.1	5.1–20.1	4.19 × 10^−11^	3834	262,425	—	36.4%	25.3%
	Colon cancer	7.5	3.3–17.0	1.89 × 10^−6^	2793	263,466	—	17.9%	12.7%
	Rectal cancer	12.4	4.4–34.8	1.79 × 10^−6^	1082	260,587	—	16.1%	9.9%
	Brain cancer (glioma)	8.9	2.2–36.2	2.19 × 10^−3^	702	358,789	—	13.4%	10.7%
*PMS2* p.Pro246Cysfs*3 Pop carrier freq: 0.234%	Endometrial cancer	9.9	4.9–19.8	1.30 × 10^−10^	923	115,104	0.99	NA	14.8%
	Colorectal cancer	3.6	2.2–5.9	7.64 × 10^−7^	3834	262,425	—	12.9%	9.0%
	Colon cancer	3.8	2.2–6.6	1.7 × 10^−7^	2793	263,466	—	9.2%	6.5%
	Rectal cancer	2.3	0.8–6.9	1.2 × 10^−1^	1082	260,587	—	3.0%	1.9%
	Ovarian cancer	3.9	1.3–11.9	1.53 × 10^−2^	779	121,299	—	NA	3.5%
*PMS2* p.Met1? Pop carrier freq: 0.092%	Endometrial cancer	7.5	2.4–23.5	5.53 × 10^−4^	923	115,104	0.99	NA	11.3%
	Colorectal cancer	2.2	0.94–5.3	7.01 × 10^−2^	3834	262,425	—	8.0%	5.5%
	Colon cancer	2.7	1.1–6.8	3.7 × 10^−2^	2793	263,466	—	6.4%	4.6%
	Rectal cancer	0.9	0.13–6.7	5.4 × 10^−1^	1082	260,587	—	1.2%	0.8%
	Ovarian cancer	6.6	1.8–24.3	4.16 × 10^−3^	779	121,299	—	NA	6.0%

F, females; Pop carrier freq, population-based carrier frequency; LS, Lynch syndrome; M, males; NA, not applicable.

Cancer risks are calculated by using lifetime cancer risk at age 75 obtained from the Icelandic Cancer Registry^18^.

In total 16 cancers were tested for each of the mutations. Significant *P*-value<3.13 × 10^−3^ to correct for multiple tests. Info represents the imputation quality of the variant. *P*-values were obtained by logistic regression analysis.

**Table 6:** Mismatch repair gene variants of uncertain significance and associated colorectal cancer risk in the Icelandic population.

**Gene**	**Hg38 location**	IHC stains in CRC pts with variant*	**Protein change**	**InSiGHT classification**	**Carrier frequency (%) in controls**	**Odds ratio for CRC**	**95% Confidence interval**	***P*****-value**	**Info**
*MLH1*									
chr3:36993584		Normal (1)	p.Glu13Lys	Class 3	0.06	0.94	0.22–4.12	0.94	0.96
chr3:36993612		*MLH1*-hm (1)	p.Gly22Ala	Class 3	0.07	0.78	0.33–1.88	0.58	0.99
chr3:37017517		Normal (2)	p.Glu268Gln	NK	0.08	1.60	0.51–5.07	0.42	0.95
chr3:37017536		None	p.Lys274Arg	NK	0.22	0.78	0.33–1.88	0.58	0.91
*MSH2*									
chr2:47429940		Normal (4)	p.Glu425.=	Class 3†	0.26	0.62	0.26–1.45	0.27	0.99
chr2:47445555		Normal (1)	p.His428Gln	NK	0.07	1.85	0.65–5.25	0.25	1.00
chr2:47476525		Normal (7), *MLH1*-hm (1)	p.Val722Ile	Class 3†	0.74	0.79	0.48–1.30	0.36	0.98
chr2:47476578		Normal (15), *MLH1*-hm (1), MSH6 miss (1)‡	c.2210+7G>T Splicing	NK	1.94	0.68	0.50–0.93	0.02	0.99
*MSH6*									
chr2:47783306		Normal (5)	p.Ala25Ser	Class 3	0.51	1.04	0.61–1.76	0.89	0.96
chr2:47796058		Normal (5) *MLH1*-hm (1)	p.Met208Val	Class 3	0.50	1.39	0.87–2.23	0.17	0.98
chr2:47801021		Normal (2)	p.Lys1013Thr	VUS on NCBI†	0.55	0.72	0.42–1.22	0.22	1.00
chr2:47803464		Normal (3)	p.Pro1073Ser	Class 3	0.1	1.36	0.47–3.93	0.57	1.00
chr2:47803506		Normal (14) *MLH1*-hm (2) MLH1 miss (1)§	p.Pro1087Ser	Class 3†	1.21	0.85	0.59–1.22	0.38	1.00
chr2:47806495		Normal (3)	p.Ile1283dup	NK	0.34	1.13	0.61–2.09	0.69	1.00
*PMS2*									
chr7:6009009		Normal (1) *MLH1*-hm (1)	p.Ala4Gly	NK	0.04	2.64	0.62–11.2	0.19	0.98
chr7:6006003		Normal (17) *MLH1*-hm (1) *PMS2* fs (1)	p.Ile18Val	Class 3†	1.73	0.90	0.67–1.21	0.47	0.99
chr7:6002515		None	p.Val159Met	Class 3	0.05	0.96	0.15–6.31	0.96	0.96
chr7:5995539		None	p.Ala300Pro	NK	0.03	0.02	0.00–8.04	0.19	0.98
chr7:5989940		Normal (1)	p.Asn335Ile	VUS on NCBI	0.03	1.38	0.24–7.97	0.72	0.93
chr7:5987328		Normal (5) *MLH1*-hm (1)	p.His479Gln	Class 3	0.30	1.12	0.58–2.15	0.75	0.98
chr7:5987315		None	p.Pro484Thr	NK	0.04	0.02	0.00–4.18	0.15	0.95
chr7:5987213		Normal (6) *MLH1*-hm (1)	p.Glu518Lys	NK	0.33	1.62	0.95–2.77	0.08	0.99
chr7:5986784		Normal (3)	p.Glu661Lys	NK	0.29	1.04	0.52–2.06	0.92	0.97

CRC, colorectal cancer; fs, frameshift; IHC, immunohistochemistry; miss, missing on stains; MLH1-hm ,*MLH1-*hypermethylated; NCBI, NK=not known; pts, patients; seq, sequenced; VUS, variants of unknown significance. Info represents the imputation quality of the variant. *P*-values were obtained by logistic regression analysis.

^*^This column displays patients in the CRC cohort with the variant (either by sequencing or imputation) and their tumour MMR stain (# patients in parenthesis).

^†^These variants can be reclassified as benign variants based on the population frequency and low odds ratio for CRC.

^‡^Patient #509 with MSH6 absent on MMR tumour immunohistochemistry with possible double somatic hit (see Table 4).

^§^Patient #506 with MLH1/PMS2 absent on MMR tumour immunohistochemistry with double somatic hit (see Table 4).
