# Supplementary material for: Comprehensive population-wide analysis of Lynch syndrome in Iceland reveals founder mutations in MSH6 and PMS2
Source: Nat Commun. 2017 May 3;8:14755. doi: 10.1038/ncomms14755 (PMC5418568; doi:10.1038/ncomms14755)
Supplement: Supplementary Information — Supplementary Figures and Supplementary Tables [file ncomms14755-s1.pdf]

Supplementary Figure 1

A - *MSH6* p.Val282Thrfs*10:

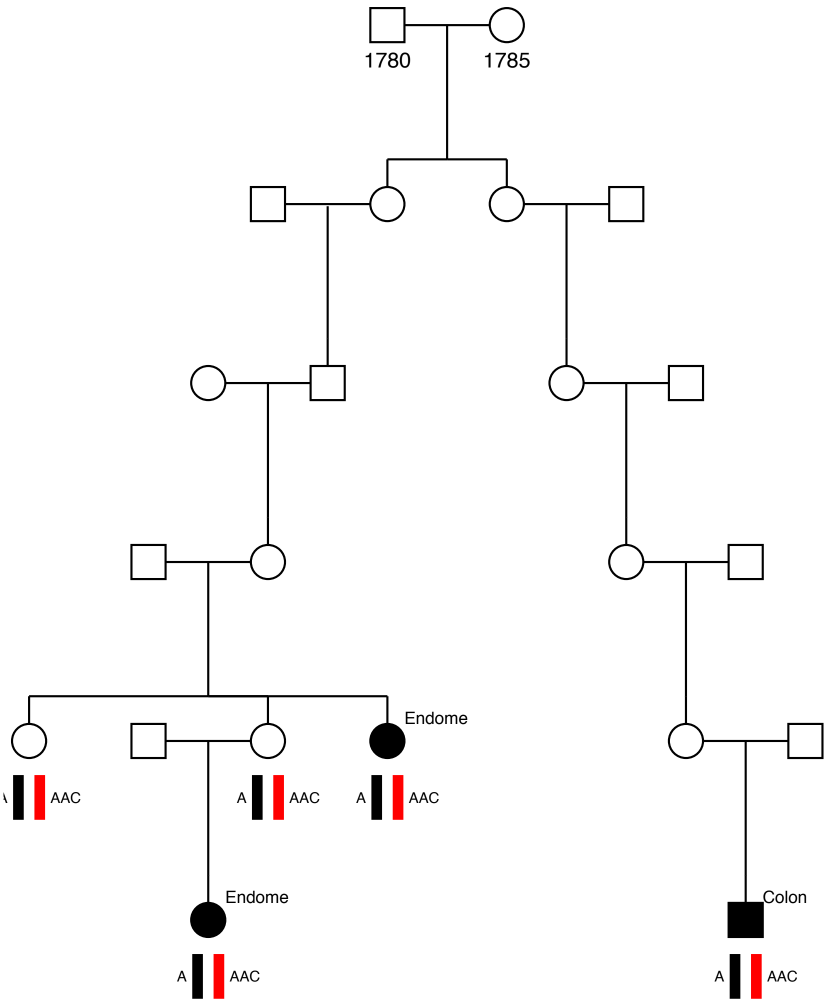

B - *MSH6* p.Phe1088Leufs*5:

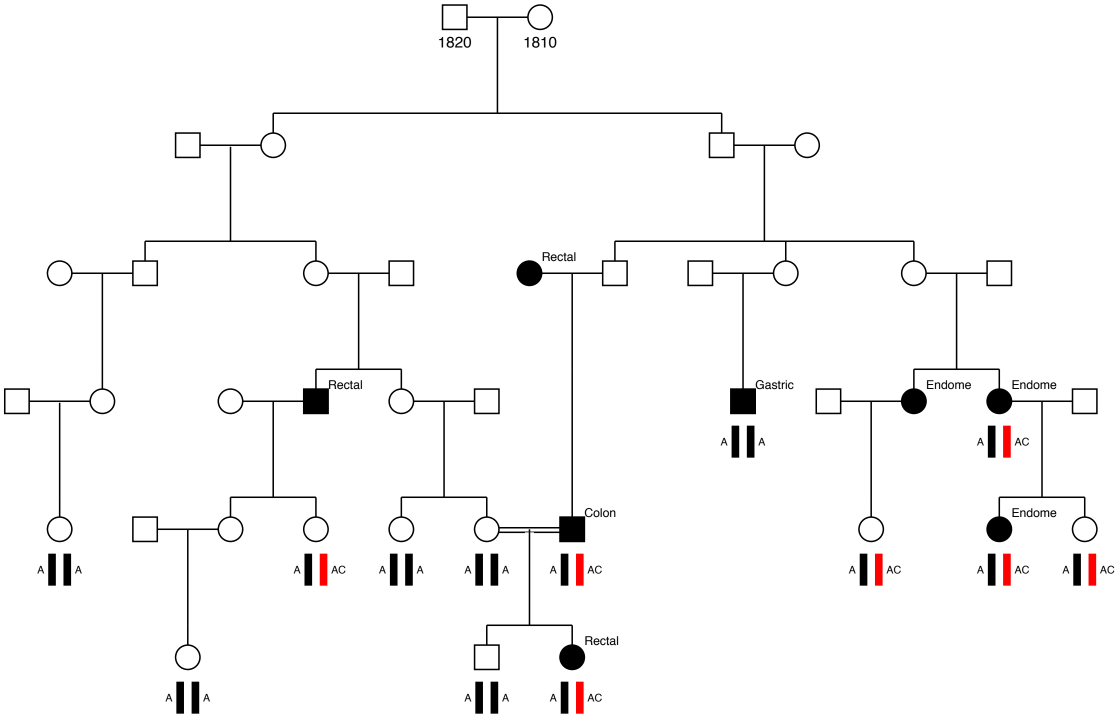

C - *MSH6* p.Arg1172Lysfs*5:

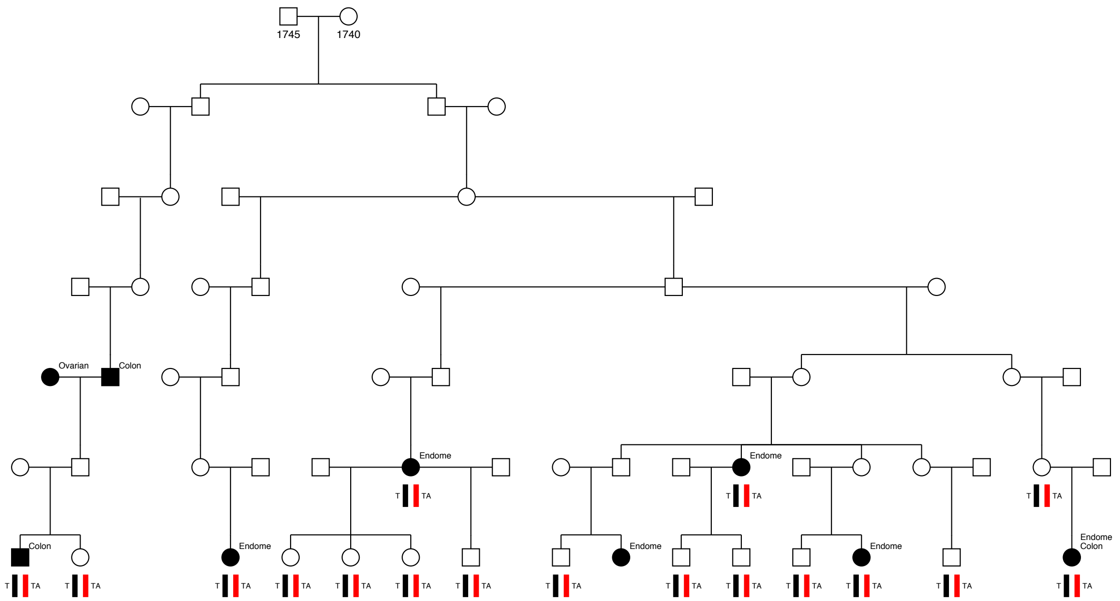

D - *MSH2* p.Tyr815*:

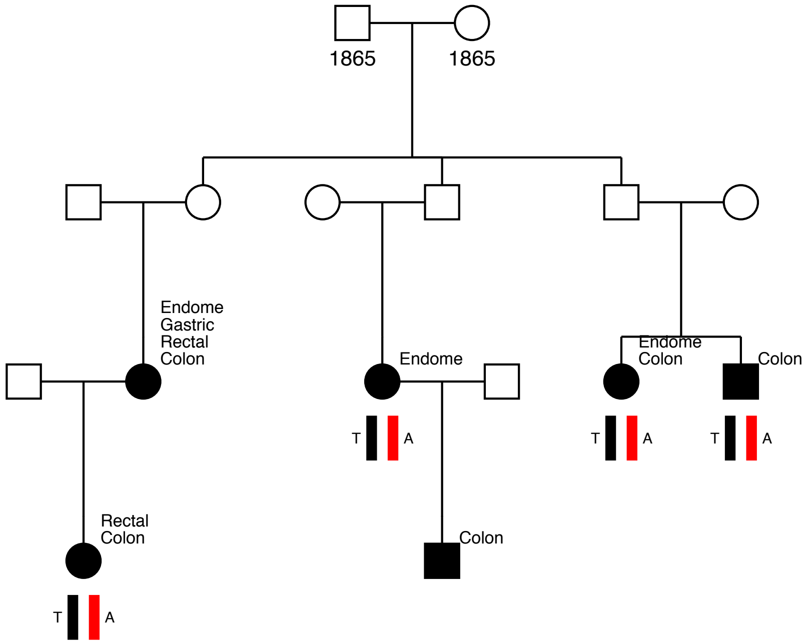

Families with private mutations. The wild-type allele (black) and haplotype allele linked to the mutation (red) are shown. The genotyped individuals are shown with letters representing the hotspot. Black circles/squares represent cancer cases. Panel A shows the family carrying *MSH6* p.Val282Thrfs*10 (AAC), panel B the family carrying *MSH6* p.Phe1088Leufs*5 (AC), panel C the family carrying *MSH6* p.Arg1172Lysfs*5 (TA) and panel D the family carrying *MSH2* p.Tyr815* (A).

Supplementary Table 1: Validation of imputation through direct genotyping of imputed heterozygous carriers and imputed wild type homozygotes

	***PMS2* p.Pro246Cysfs*3 (Freq=0.234%)** Imputation		***MSH6* p.Leu585Pro (Freq=0.08%)** Imputation		***PMS2* p.Met1? (Freq=0.092%)** Imputation	
**Direct genotyping**	wt	het	wt	het	wt	het
wt[a]	15,540	0	130	0	2963	0
het[b]	0	49	1	62	1	14

Freq=population-based carrier frequency; het=heterozygous; wt=wildtype.

Counts correspond to individuals where direct genotyping was successful; genotyping success rate was 91% for *PMS2* p.Pro246Cysfs*3, 92% for *MSH6* p.Leu585Pro and 97% for *PMS2* p.Met1?.

[a]Homozygous wild type carriers

[b]Heterozygous carriers

Supplementary Table 2: Patient and tumor characteristics in Lynch syndrome cases

Study ID	Age, sex	Location	Histology, grade	Stage	Absent stains on IHC	Germline mutation
339	67 M	R	Mucinous, G NK	I	MLH1/PMS2	*MLH1* translocation
472	79 M	R	Adenocarcinoma, G2	IIIB	MSH6	*MSH6* p.Val282Thrfs*10
357	68 M	L	Adenocarcinoma, G3	IIIC	MSH6	*MSH6* p.Phe1088Leufs*5
172	56 M	R	Adenocarcinoma, G2	IIIB	MSH6	*MSH6* p.Arg1172Lysfs*5
427	65 M	R	Mucinous, G2	IVA	PMS2	*PMS2* p.Glu705Lys
1057	55 M	R	Adenocarcinoma, G3	IVA	PMS2	*PMS2* p.Asn71Aspfs*4
960	75 F	R	Adenocarcinoma, G2	IIA	MSH6	*MSH6* p.Leu585Pro
736	60 M	L	Mucinous, G2	IIA	MSH6	*MSH6* p.Leu585Pro
438	55 M	Rec	Mucinous, G2	IIA	MSH6	*MSH6* p.Leu585Pro
455	74 F	L	Adenocarcinoma, G3	IIA	MSH6	*MSH6* p.Leu585Pro
508	47 M	R	Adenocarcinoma, G3	IIA	MSH2/MSH6	*MSH6* p.Leu585Pro
900	41 M	Rec	Adenocarcinoma, G2	I	Normal (MSH6 5%)	*MSH6* p.Leu585Pro
786*	63 M	L	Mucinous, G2	IIC	Normal (MSH6 weak, 1-5%)	*MSH6* p.Leu585Pro
30*	57 M	L	Mucinous, G2	IIA	Normal (MSH6 weak, 5%)	*MSH6* p.Leu585Pro
94*	72 F	L	Adenocarcinoma, G2	IIA	Normal (MSH6 weak, 20-30%)	*MSH6* p.Leu585Pro
1245	40 F	R	Adenocarcinoma, G2	IIA	MSH6/PMS2	*PMS2* p.Pro246Cysfs*3
895	30 F	Rec	Adenocarcinoma, G2	I	PMS2	Presumed *PMS2* p.Pro246Cysfs*3
667	86 M	L	Mucinous, G NK	IIB	MSH2/6/PMS2	*PMS2* p.Pro246Cysfs*3
912	55 M	R	Adenocarcinoma, G2	I	PMS2	*PMS2* p.Pro246Cysfs*3
923	63 M	R	Adenocarcinoma, G2	IIA	PMS2	*PMS2* p.Pro246Cysfs*3
363	79 M	R	Adenocarcinoma, G2	IIA	PMS2	*PMS2* p.Pro246Cysfs*3
268	52 M	R	Adenocarcinoma, G2	I	PMS2	*PMS2* p.Pro246Cysfs*3
231	62 M	L	Adenocarcinoma, G2	IIA	PMS2	*PMS2* p.Pro246Cysfs*3

210	72 M	R	Adenocarcinoma, G2	IIC	PMS2	*PMS2* p.Pro246Cysfs*3
99	50 M	R	Adenocarcinoma, G2	IIA	PMS2	*PMS2* p.Pro246Cysfs*3
1250*	59 M	L	Adenocarcinoma, G2	I	Normal	*PMS2* p.Pro246Cysfs*3
873*	64 M	Rec	Adenocarcinoma, G2	IIA	Normal	*PMS2* p.Pro246Cysfs*3

*These cases had tumor sent for ColoSeq (Results in Table 4).

F=female; G=grade; IHC=immunohistochemistry; L=left; LS=Lynch syndrome; M=male, NK=not known; R=right; Rec=rectum

Supplementary Table 3: Haplotype analysis in carriers of the *PMS2* frameshift mutation

	rs2228007 chr7:5987234	rs12702460 chr7:5993117	rs7788441 chr7:5996313	rs7805798 chr7:5996374	rs7793254 chr7:5997171	Indel chr7:5997387	rs12702463 chr7:5998548	rs6949598 chr7:5998852
Carrier 1	C	A	G	T	G	Yes	C	A
Carrier 2	C	A	G	T	G	Yes	C	A
Carrier 3	C	A	G	T	G	Yes	C	A
Carrier 4	C	A	G	T	G	Yes	C	A
Carrier 5	C	A	G	T	G	Yes	C	A
Carrier 6	C	A	G	T	G	Yes	C	A
Carrier 7	C	A	G	T	G	Yes	C	A
Carrier 8	C	A	G	T	G	Yes	C	A
Carrier 9	C	A	G	T	G	Yes	C	A
Carrier 10	C	A	G	T	G	Yes	C	A
Carrier 11	C	A	G	T	G	Yes	C	A
Carrier 12	C	A	G	T	G	Yes	C	A
MAF*	C=3.39%	A=15.82%	A=31.16%	T=15.70%	G=26.88	0.117%	C=15.75%	A=15.85%

Hg38 location displayed. Haplotype obtained from Clendenning 2008.[20]

*Minor allelic frequency in the Icelandic population.

Supplementary Table 4: Icelandic Lynch syndrome mutations and cancer risks

	Cancer type	Odds ratio	95% CI	p-value	Cases	Controls	Info
	Bladder cancer	1.7	0.35-8.1	$5.12x10^{-1}$	1888	291,370	0.92
	Breast cancer	0.96	0.26-3.6	$9.48x10^{-1}$	5727	312,993	
MSH6 p.Leu585Pro							
Freq: 0.080%[1)]	Esophageal cancer	1.3	0.03-57.8	$8.86x10^{-1}$	620	205,517	
	Gallbladder/bile duct cancer	3.0	0.09-97.4	$5.3x10^{-1}$	349	233,076	
	Gastric cancer	2.1	0.5-8.6	$3.1x10^{-1}$	2671	240,521	
	Liver cancer	5.4	0.43-67.6	$1.9x10^{-1}$	273	301,484	
	Ovarian cancer	3.1	0.38-25.0	$2.95x10^{-1}$	779	121,299	
	Pancreatic cancer	2.4	0.26-22.5	$4.4x10^{-1}$	1177	200,727	
	Prostate cancer	1.5	0.49-4.5	$4.8x10^{-1}$	5274	97,905	
	Testicular cancer	4.4	0.37-52.5	$2.44x10^{-1}$	300	151,991	
	Bladder cancer	1.0	0.35-2.9	$9.9x10^{-1}$	1888	291,370	0.99
	Breast cancer	1.9	1.1-3.4	$3.10x10^{-2}$	5727	312,993	
	Brain cancer (glioma)	2.0	0.45-9.3	$3.6x10^{-1}$	702	358,789	
PMS2 p.Pro246Cysfs*3	Esophageal cancer	2.2	0.52-9.1	$2.86x10^{-1}$	620	205,517	
Freq: 0.234%[2)]	Gallbladder/bile duct cancer	0.49	0.006-42.7	$7.6x10^{-1}$	349	233,076	
	Gastric cancer	1.7	0.72-4.0	$2.2x10^{-1}$	2671	240,521	
	Liver cancer	1.1	0.02-51.7	$9.6x10^{-1}$	273	301,484	
	Prostate cancer	1.3	0.69-2.5	$4.1x10^{-1}$	5274	97,905	
	Testicular cancer	1.5	0.18-12.6	$7.0x10^{-1}$	300	151,991	
	Bladder cancer	0.53	0.085-3.27	$4.9x10^{-1}$	1888	291,370	0.99
PMS2 p.Met1?	Brain cancer (glioma)	0.60	0.007-47.8	$8.2x10^{-1}$	702	358,789	
Freq: 0.092%[3)]	Breast cancer	0.84	0.29-2.5	$7.6x10^{-1}$	5727	312,993	

Gallbladder/bile duct cancer	0.78	0->100	$9.5x10^{-1}$	349	233,076
Gastric cancer	2.4	0.88-6.7	$8.54x10^{-2}$	2671	240,521
Liver cancer	3.6	0.33-38.2	$3.0x10^{-1}$	273	301,484
Pancreatic cancer	1.4	0.21-8.82	$7.5x10^{-1}$	1177	200,727
Prostate cancer	0.83	0.28-2.5	$7.4x10^{-1}$	5274	97,905
Testicular cancer	7.4	0.8-68.4	$7.8x10^{-2}$	300	151,991

CI=confidence interval; Freq=frequency; LS=Lynch syndrome. Info represents the imputation quality of the variant. p-values were obtained by logistic regression analysis.

1) No association seen with adrenal gland, renal pelvis or ureteral cancer.

2) No association seen with adrenal gland, renal pelvis, ureteral and pancreatic cancer.

3) No association seen with adrenal gland, esophageal, renal pelvis and ureteral cancer.

In total 16 cancers were tested for each of the mutations. Significant p-value $< 3.13x10^{-3}$ to correct for multiple tests.

Supplementary Table 5: Primer sequences used for Sanger sequencing of individual variants

Gene	Protein change	Hg38 location	Forward	Reverse
MSH2	p.Tyr815*	chr2:47478506	CTGCCTTGGCCAATCAGATA	AAGGCAATTACTGATGATTTCAA
MSH6	p.Val282Thrfs*10	chr2:47798826	TGACATTGGTGGCTCTGATG	CTTCCGCTTTCGAGCAACTT
MSH6	p.Leu585Pro	chr2:47799737	GATGATCGCCATTGTTCGAG	GCCGGGTATCAGACCTTCCT
MSH6	p.Phe1088Leufs*5	chr2:47803500	GCTGATAAAACCCCCAAACG	TTTGCCATTTTCCTGCTCCT
MSH6	p.Arg1172Lysfs*5	chr2:47804984	GCCTCTTGTCTCTCTTAGCCTCAA	TTCTGTCTGAGGCACCAAGTCT
PMS2	Glu705Lys	chr7:5982885	GGGGAGTCTGGGAATGAACA	GAGCCAAGATTGTGCCATTG
PMS2	p.Pro246Cysfs*3	chr7:5997387	AAAAAGATTATGCAGAGCATCG	TCAGTTGCAAAGCCTCATTC
PMS2	p.Asn71Aspfs*4	chr7:6004007	CCCGAAAGCCAAAAGTTTCA	TGATAGCATGGGTCCGTTTTT
PMS2	p.Met1?	chr7:6009018	ATGCCGTGGGTCTCAAAGAG	GGAGGGAACTTTCCCAGTCC
