# Supplementary material for: Comprehensive population-wide analysis of Lynch syndrome in Iceland reveals founder mutations in MSH6 and PMS2
Source: Nat Commun. 2017 May 3;8:14755. doi: 10.1038/ncomms14755 (PMC5418568; doi:10.1038/ncomms14755)
Supplement: Peer Review File [file ncomms14755-s2.pdf]

Editorial Note: this manuscript has been previously reviewed at another journal that is not operating a transparent peer review scheme. This document only contains reviewer comments and rebuttal letters for versions considered at Nature Communications.

REVIEWERS' COMMENTS:

Reviewer #3 (Remarks to the Author):

The revised manuscript has addressed most of the reviewers' comments and provided sufficient clarifications. I have no further comment. It represents a comprehensive colorectal cancer cohort from the Icelandic population with detailed characterisation for Lynch Syndrome mutations, thus of significance and interest for readers of Nature Communications.
